# Active-site engineering of ω-transaminase from *Ochrobactrum anthropi* for preparation of L-2-aminobutyric acid

**DOI:** 10.1186/s12896-021-00713-7

**Published:** 2021-09-25

**Authors:** Zhiwei Zhang, Yang Liu, Jing Zhao, Wenqiang Li, Ruiwen Hu, Xia Li, Aitao Li, Yaping Wang, Lixin Ma

**Affiliations:** State Key Laboratory of Biocatalysis and Enzyme Engineering, Hubei Collaborative Innovation Center for Green Transformation of Bio-Resources, Hubei Key Laboratory of Industrial Biotechnology, College of Life Sciences, Hubei University, 368 Youyi Road, Wuchang, Wuhan, 430062 China

**Keywords:** ω-transaminase, Saturation mutagenesis, L-2-aminobutyric acid, Molecular docking, L57C/M419I variant

## Abstract

**Background:**

The unnatural amino acid, L-2-aminobutyric acid (L-ABA) is an essential chiral building block for various pharmaceutical drugs, such as the antiepileptic drug levetiracetam and the antituberculosis drug ethambutol. The present study aims at obtaining variants of ω-transaminase from *Ochrobactrum anthropi* (OATA) with high catalytic activity to α-ketobutyric acid through protein engineering.

**Results:**

Based on the docking model using α-ketobutyric acid as the ligand, 6 amino acid residues, consisting of Y20, L57, W58, G229, A230 and M419, were chosen for saturation mutagenesis. The results indicated that L57C, M419I, and A230S substitutions demonstrated the highest elevation of enzymatic activity among 114 variants. Subsequently, double substitutions combining L57C and M419I caused a further increase of the catalytic efficiency to 3.2-fold. This variant was applied for threonine deaminase/OATA coupled reaction in a 50-mL reaction system with 300 mM L-threonine as the substrate. The reaction was finished in 12 h and the conversion efficiency of L-threonine into L-ABA was 94%. The purity of L-ABA is 75%, > 99% *ee*. The yield of L-ABA was 1.15 g.

**Conclusion:**

This study provides a basis for further engineering of ω-transaminase for producing chiral amines from keto acids substrates.

**Supplementary Information:**

The online version contains supplementary material available at 10.1186/s12896-021-00713-7.

## Background

L-ABA is an essential chiral building block for various pharmaceutical drugs, such as the antiepileptic drug levetiracetam and the antituberculosis drug ethambutol, etc. [1–3]. The Asymmetric synthesis of L-ABA with both chemocatalytic and biocatalytic strategies has been developed and the biocatalytic methods have attracted growing attention due to environmental and social demands for green processes in the chemical industry recently [4]. The most intensively studied biocatalytic methods for the synthesis of L-ABA focus on enzyme-based kinetic resolution of (R, S)-2-aminobutyric acid and amination of α-ketobutyric acid using L-amino dehydrogenase [5] or transaminase [6]. In the reductive amination process with dehydrogenase using L-threonine as the precursor, formate dehydrogenase (FDH) is added to regenerate NADH. However extra NAD^+^ must be added to the mixture during the reaction in a timely fashion to guarantee the complete bioconversion of L-threonine to L-ABA [7]. Hence, the high cost of NAD^+^ limited its application in the industry.

On the other hand, transaminases are pyridoxal-5′ phosphate (PLP)-dependent enzymes [8] that are involved in reversible transfer of amino groups from amino donors to carbonyle of amino receptors. Transaminases display high reaction rates, broad substrate spectrum, and no requirement of cofactor regeneration [2], which render these enzymes attractive for industrial process development [4, 9]. In 2001, Fotheringham’s group developed a three-enzyme system including threonine deaminase, tyrosine aminotransferase, and acetolactate synthase to produce L-ABA with L-aspartic acid and L-threonine as starting materials. L-threonine was converted by threonine deaminase of *E. coli* to obtain α-ketobutyric acid, which in turn, converted to L-ABA using the aromatic transaminase of *E. coli* encoded by *tyr*B, with L-aspartate as the amino donor. Meanwhile, acetolactate synthase from *Bacillus subtilis* 168 eliminates pyruvate and alanine, which are the by-products of aspartate transamination. The overall yield of L-ABA was 54% [10]. Zhu et al. optimized this method by introducing alanine racemase and D-amino acid oxidase into the system [11]. In 2009, Shin et al. chose a ω-transaminase (ω-TA) from *Vibrio fluvialis* JS17 to catalyze the asymmetric synthesis of L-ABA using 2-oxobutyric acid and benzylamine as starting materials in an extractive biphasic reaction system [1]. In 2018, Heuson et al. realized both enantiomers of ABA were efficiently prepared in an equilibrium shifted reaction using glutamine as amine donor [8]. In 2010, Park et al. established a cost-effective TD/ω-TA coupled one-pot method to produce L-ABA. L-threonine is deaminized with threonine deaminase (TD, EC4.2.1.6) encoded by *ilv*A gene of *E. coli* to generate α-ketobutyric acid in the first step. Next, L-ABA was synthesized through reductive amination with benzylamine as amino donor. (S)-selective ω-TA from *Ochrobactrum anthropi* (OATA) was employed to catalyze this process. The conversion yield reached 91% with 10 mM L-threonine and 20 mM benzylamine. Park modified this process by using isopropylamine as amino donor (2013). OATA has exceptionally high activity for isopropylamine and the deamination product of isopropylamine is acetone, a non-reactive amino acceptor easily removable owing to high volatility. Therefore, this reaction overcame the unfavorable equilibrium. Park’s study demonstrated a TD/OATA coupled reaction using L-threonine (100 mM) and isopropylamine (2 Equiv.) to generate L-ABA and gained 99% conversion yield with 99% ee in 1 h. Preparative scale synthesis with L-threonine (1.79 g, 15 mmol) and isopropylamine (1.94 mL, 22.5 mmol) in a 50-mL reaction system, conversion efficiency of over 99% was attained within 12 h [12].

In the TD and OATA cascade reaction, the reaction catalyzed by TD was much faster than that of OATA. Asymmetric synthesis of chiral amines using OATA has suffered from an unfavorable reaction equilibrium when a high concentration of substrate was used [13–15]. Therefore, OATA determines the conversion yield of the whole process. To overcome this limitation, creating OATA variants with high turnover efficiency for asymmetric amination of α-ketobutyric acid is necessary [16–18]. In the present study, we performed substrate docking simulations to identify key active-site residues around α-ketobutyric acid and performed saturation mutagenesis to these amino acid residues. The result indicated that L57C, M419I and A230S substitutions demonstrated the highest elevation of enzymatic activity and double substitutions combining L57C and M419I caused a further increase of the catalytic efficiency. This variant was applied for TD/OATA coupled reaction in a 50-mL reaction system and demonstrated higher conversion efficiency in comparison with wild-type OATA.

## Results

### Molecular docking and engineering of OATA

**Fig. 1:**
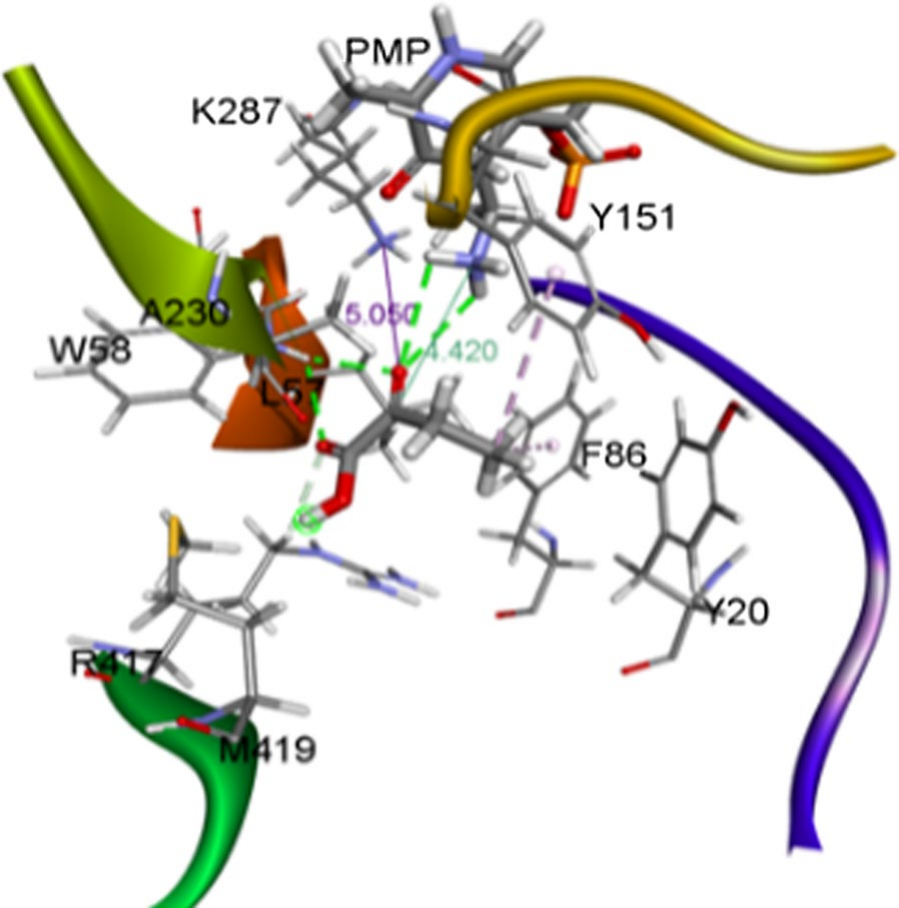
Model of α-ketobutyric acid docking into the active site of OATA. pyridoxamine 5′-phosphate (PMP) and α-ketobutyric acid are shown in thick sticks. The hydrogen bonds and Pi-Alkyl interactions are indicated with green and pink dotted lines, respectively. The Cα − NPMP and Oβ − NK287 distances are designated by dark green and purple lines, respectively

Substrate docking simulation using α-ketobutyric acid as the ligand is shown in Fig. 1 (Additional file 2: Table S1). The active site harboring an inward-point arginine (R417) was used for docking simulation [24]. The structure of OATA with pyruvic acid as substrate was used as the template. When R417 adopts an outward conformation, the large pocket becomes too contracted to recognize the carboxyl group. On the contrary, the large pocket is able to accumulate a bigger group, such as keto acid group of α-ketobutyric acid when R417 adopts inward conformation. Therefore, we chose an inward conformation of R417 for docking simulation. For the docking simulation, the ethyl group of α-ketobutyric acid is located in a small pocket while the carboxyl group is located in a large pocket. The ethyl group was wrapped by amino acid residues including Y20, F86, Y151, and A230. It is worth noting that W58 and R417 in the large pocket formed multiple hydrogen bonds with the carbonyl oxygen and carboxyl group of the substrate, which implied the importance of those two amino acids.

Based on the result of docking simulation, 6 amino acid residues, consisting of Y20, L57, W58, G229, A230 and M419, lying within a 5 Ǻ- distance from the bound α-ketobutyric acid were selected for saturation mutagenesis and all the 114 possible variants with a single mutation were generated. The activity of all OATA variants for α-ketobutyric acid was tested with thin layer chromatography (TLC). The result of TLC indicated that L57A, L57C, A230S, M419I, M419W, M419C, and M419A displayed an obvious increase in the conversion efficiency of α-ketobutyric acid to L-ABA while M419T, and M419V showed a slight elevation in comparison with wild-type OATA (Additional file 1: Figure S1). Consistent with the docking model, W58 is an important amino acid residue and all the substitutions of W58 failed to improve the activity of OATA.

**Fig. 2:**
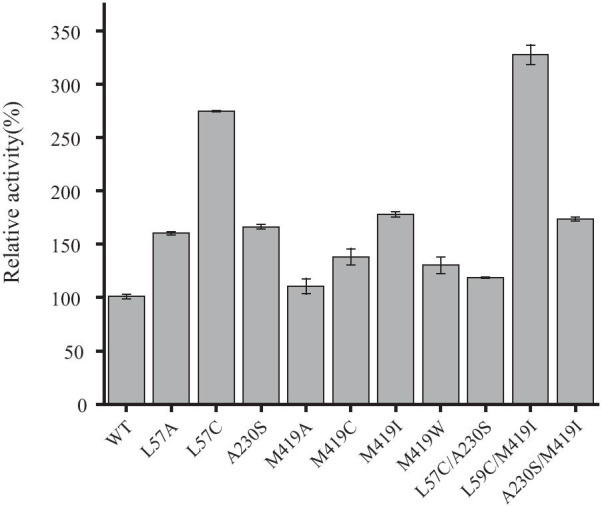
Comparison of enzyme activities of mutants relative to wild type. The reaction mixture (100 μL) contained 0.5 mM PLP, OATA (0.25 mg/mL), 300 mM α-ketobutyric acid, 450 mM isopropylamine and 50 mM phosphate buffer (pH 7.5). The reaction was carried out at 37 °C for 30 min

Mutants showing obviously improved activity were purified (Additional file 1: Figure S2) and their activities were analyzed with HPLC. The result indicated that three variants L57C, M419I and A230S demonstrated the most obvious improvement, which were 2.7, 1.8 and 1.7-fold, respectively (Fig. 2). To achieve further improvements, these three mutations were combined to generate three double mutants, L57C/A230S, L57C/M419I and A230S/M419I. Among them, OATA_L57C/M419I_ showed the most obvious improvement. Its catalytic efficiency increased 3.2-fold in comparison with wild-type OATA. Both L57C/A230S and A230S/M419I variants demonstrated an elevation of specific activity compared with wild-type, but reduced activity against single mutants (Fig. 2).

### Kinetic analysis of L57C/M419I variant

The kinetic parameters of the wild-type OATA and OATA_L57C/M419I_ was investigated (Table 1, Additional file 1: Figure S5, Additionl file 2: Table S2). The K_m_ of the mutant was similar to that of the wild-type enzyme. On the contrary, the catalytic turnover increased 2.2-fold in comparison with the wild-type enzyme, leading to a 2.3-fold increase in the *k*_cat_/K_m_ for the transaminase. This result indicated that the double mutation affected the catalytic efficiency of OATA.

**Table 1:** Apparent kinetic parameters of the wild-type OATA and OATA_L57C/M419I_ for α-ketobutyric acid

	Wild-type	L57C/M419I
K_m_ (mM)	266 ± 34	260 ± 6
*k*_cat_ (s^−1^)	5.1 ± 0.3	11.5 ± 0.3
*k*_cat_/K_m_ (M^−1^ s^−1^)	19 ± 3	44 ± 1

The reaction was carried out in a 100-μL mixture including 0.5 mM PLP, 0.25 mg/mL OATA, 50–650 mM α-ketobutyric acid at a fixed concentration of isopropylamine (1 M) and 50 mM phosphate buffer (pH 7.5). The mixture was incubated at 37 °C for 30 min

### Mechanism analysis for the improved activity of OATA_L57C/M419I_

**Fig. 3:**
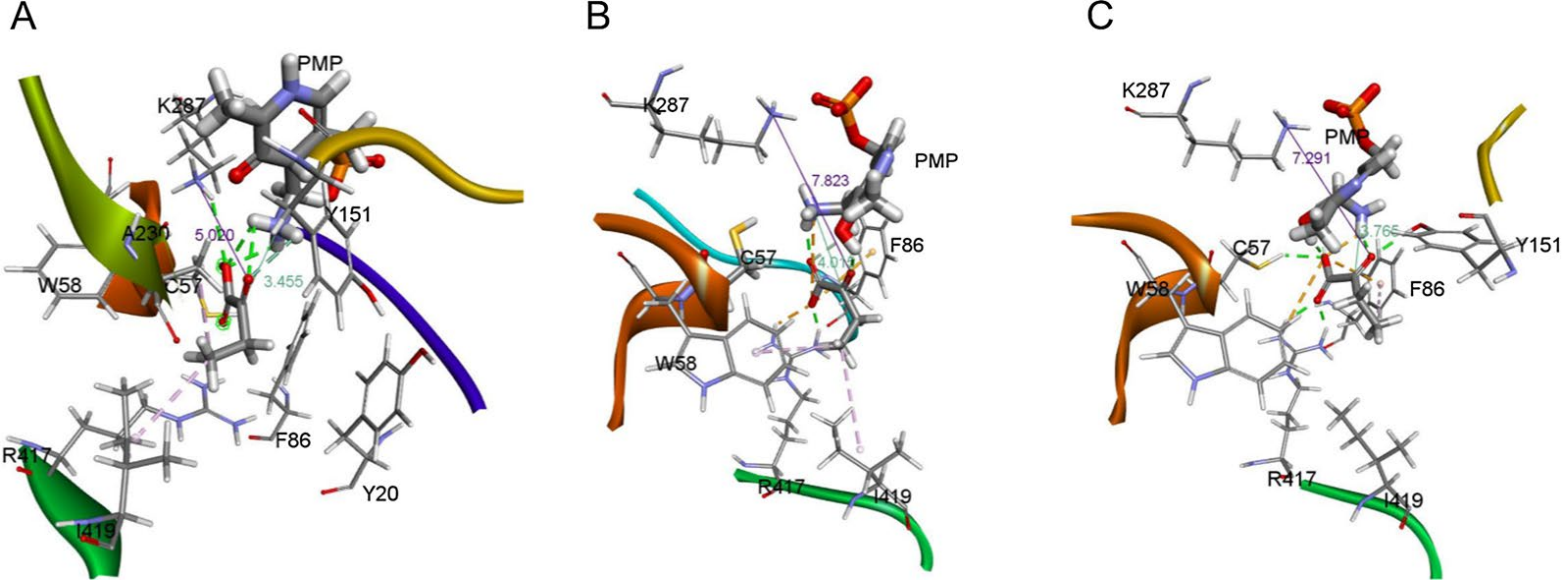
The mechanism analysis of mutations. **A** Docking models of the OATA_L57C/M419I_ variant using α-ketobutyric acid as the ligand. **B** and **C** Molecular dynamic simulations of the OATA_L57C/M419I_ variant using α-ketobutyric acid as the ligand. PMP and α-ketobutyric acid are shown in thick sticks. The hydrogen bonds and salt bridge are indicated with green dotted lines and orange dotted lines, and alkyl interactions are labeled with pink dotted lines. The C_α_ − N_PMP_ and O_β_ − N_K287_ distances are designated by dark green and purple lines, respectively

In comparison with the docking location of α-ketobutyric acid in the wild-type enzyme, the substrate in the mutant active site spun clockwise and the ethyl group oriented to I419 (Fig. 3A). This translocation allowed a new hydrogen bond formed between the carbonyl oxygen of the substrate and PMP while the preexisting hydrogen bonds between W58 and the substrate were concurrently lost. Meanwhile, hydrophobic interaction formed between I419, A230, and ethyl group of the substrate attributed new forces to stabilize the substrate. The altered binding of α-ketobutyric acid in the active site of the mutant led to a significant decrease in the distance between carbonyl carbon of the substrate and the PMP from 4.420 to 3.455Ǻ, which might contribute to the increased catalytic activity.

Molecular dynamics (MD) simulations [19] is powerful in studying the effects of mutations on protein structures and the impact of protein motions on catalysis [20]. To obtain more details about the interactions between α-ketobutyric acid and the amino acid residues in the active site of the L57C/M419I variant, we performed a molecular dynamic simulation using YASARA. Before the molecular dynamic simulation, the docking simulation of α-ketobutyric acid in the active site of OATA_L57C/M419I_ was accomplished using YASARA. The conformation of the substrate with the lowest energy was chosen for molecular dynamic simulations (Fig. 3B). The result of molecular dynamic simulation indicated C57 stabilized α-ketobutyric acid in the active site and shortened the distance between the carbonyl oxygen of α-ketobutyric acid and the amino group of PMP. During the approaching of the substrate to the active site, the hydrophobic interaction between I419 and the substrate was broken, and a hydrogen bond formed between C57 and α-ketobutyric acid, resulting in the translocation of carbonyl carbon of the substrate toward PMP (Fig. 3C). The increased accessibility and stability between the substrate and the active site of the variant might lead to an improved *k*_cat_.

### The stability of the mutants

**Fig. 4:**
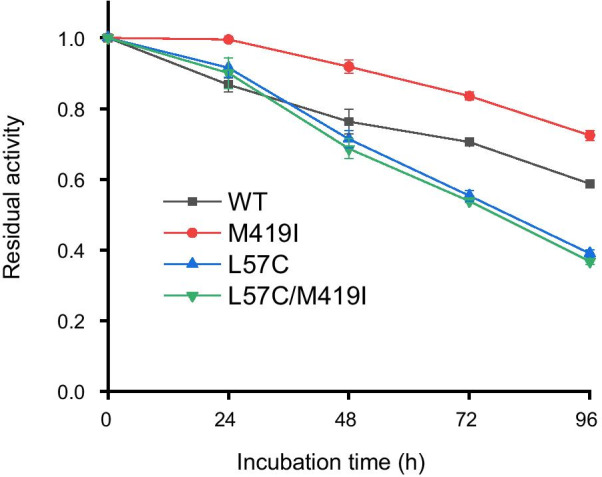
Effect of mutations on enzyme stability. Purified enzymes were incubated in 50 mM phosphate buffer (pH 7.5) with 2.5 mg/mL OATA at 37 °C. The reaction mixture (100 μL) contained 0.5 mM PLP, OATA (0.25 mg/mL), 300 mM α-ketobutyric acid, 450 mM isopropylamine and 50 mM phosphate buffer (pH 7.5). The reaction was carried out at 37 °C for 30 min

The enzyme variants need to retain the intrinsic stability of the wild-type enzyme or gain enhanced stability for their practical applications. Therefore, time-course monitoring of the enzyme activities was performed at 37 °C for 96 h in 50 mM phosphate buffer (pH7.5). M419I single mutation improved the stability of OATA (Fig. 4). This variant retained almost all activity after 24-h incubation at 37 °C and approximately 80% of its activity after 96-h treatment. On the other hand, L57C and L57C/M419I variants displayed similar thermostability as wild-type OATA in the first 48 h and became less stable than wild-type with longer treatment (Fig. 4).

### Application of OATA_L57C/M419I_ to the TD/OATA coupled reaction to generate L-ABA

**Fig. 5:**
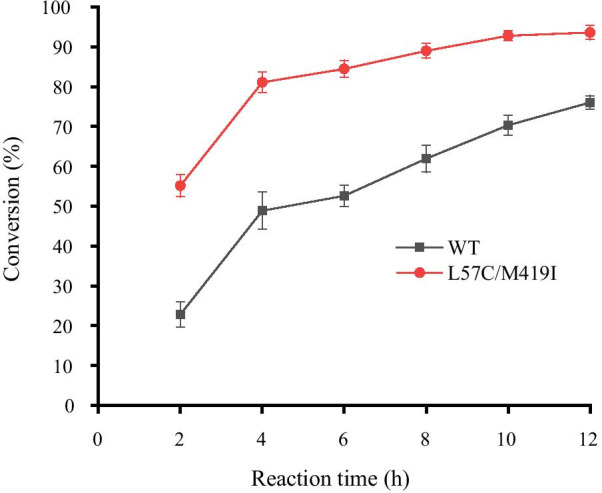
Small-scale preparation of L-ABA with OATA_L57C/M419I_ and TD. The reaction mixture (50 mL) contained 0.5 mM PLP, TD (0.2 mg/mL), OATA (1 mg/mL), 300 mM L-threonine, 450 mM isopropylamine and 50 mM phosphate buffer (pH 7.5). The reaction was carried out at 37 °C for 30 min

OATA_L57C/M419I_ was utilized for small-scale TD/OATA coupled reaction using L-threonine as the precursor (Fig. 5, Additionl file 2: Table S3). In comparison with wild-type, the conversion efficiency of the double mutant was significantly elevated. The conversion efficiency of L-threonine into L-ABA was 94% in 12 h. The purity of L-ABA was 75%, > 99% *ee*. The yield of L-ABA was 1.15 g. However, using wild-type OATA shown in our work only obtained ~ 74% completion, while the previous report reached 99% conversion efficiency [12]. We speculate the acetone or ammonia by product without volatilization could inhibit the activity of OATA.

## Discussion

With the increasing demand for chiral amines, ω- Transaminase has a broader application prospect. However, the natural ω-transaminase still displays drawbacks at stability, and catalytic efficiency. To meet the needs of industrial applications, ω-transaminase and variants with high catalytic activity were obtained through gene mining and protein engineering. The present study is aimed to elevate the catalytic activity of OATA towards α-ketobutyric acid through rational design and saturation mutagenesis. ω-transaminase carries two binding pockets, i.e. large and small pockets. The small pocket is found to display a steric constraint prohibiting entry of a substituent larger than an ethyl group, whereas the large pocket can accept a bulk substituent, such as a phenyl group through a hydrophobic interaction [21]. Previous reports indicated L57 is located in the large pocket and exhibits steric interference with the substrate substituent bound in the small pocket [22, 23]. L57A substitution removed this hindrance and resulted in a mutant with improved specificity towards a large number of bulky substrates [24–26]. In the present study, we found L57C substitution, which has never been reported previously, improved the conversion efficiency of OATA to α-ketobutyric acid. Considering L57 is located at the interface the OATA dimer, we proposed that an extra disulfide bond was formed between two monomers due to L57C mutation and increased the stability of the enzyme. The protein bearing L57C substitution was purified as described in the methods section but without the presence of β-mercaptoethanol. However, MS (mass spectrum) result indicated no novel disulfide bond was formed (data not shown). The result of molecular dynamic simulation indicated C57 formed a novel hydrogen bond with α-ketobutyric acid in the active site and shortened the distance between the carbonyl oxygen of α-ketobutyric acid and the amino group of PMP, in turn, promoted the transamination. Meanwhile, M419, a residue away from the active site, is also proved to be important to the enzymatic activity of OATA. the structure of OATA (5GHF) indicated M419 is located at the entrance of the active site (Fig. 1). Therefore, M419 interacts with α-ketobutyric acid before it enters the active site. This theory may explain why the substitutions of M419 to nonpolar amino acids, such as Ala, Val, and Ile are all able to improve the activity of OATA while all substitution of charged amino acid residues decreases its activity. Among them, M419I showed the most obvious effect. The molecular dynamic simulation demonstrated hydrophobic interaction was formed between Ile and α-ketobutyric acid, which facilitated the entrance of the substrate to the active site. Since I419 and C57 interact with α-ketobutyric acid individually during the approach of the substrate to PMP, these two substitutions have synergistic effect in improving the activity of OATA.

## Conclusion

In conclusion, the present study is another successful example for the engineering of ω-Transaminase and the result of the present study expands the pool of amino acid residues for future protein engineering of ω-TA. OATA_L57C/M419I_ showed the most obvious improvement of catalytic efficiency in comparison with wild-type OATA. In the future, we will apply this mutant to other ketone acids and ketone substrates to broaden its industrial application.

## Methods

### Strains, plasmids, medium, and reagents

*Escherichia coli* DH5α for gene cloning was from TaKaRa (China). The expression vector pET28a and *E. coli* BL21 (DE3) was from Novagene (Beijing, China). Expression vectors for the heterologous expression of TD and OATA were constructed in the present study. Briefly, the ORF of *ilv*A from *E. coli* K12 (GenBank accession number: NP_418220.1) was amplified with F1/R1 (Additional file 1: Table S1) and cloned into pET28a vector using the T5 exonuclease mediated cloning method [27]. The ORF encoding OATA (GenBank accession number: WP_011982390.1) was optimized based on the codon usage preference of *E. coli* without changing the amino acid sequence and synthesized by Sangon (China). The ORF was also cloned into the pET28a vector using the T5 exonuclease mediated cloning method. To facilitate the purification, the recombinant TD and OATA were expressed with a 6×His tag fused to their C-termini. The recombinant plasmids were identified with DNA sequencing and named pET28a-TD-His6 and pET28a-OATA-His6, respectively.

Luria–Bertani (LB) medium for the cultivation of *E. coli* was prepared as described in the Manual of Molecular Cloning [28]. L-threonine, α- ketobutyric acid, and isopropylamine were purchased from Aladdin (China). All other chemicals were analytical reagents.

### Expression and purification of recombinant TD and OATA

To induce the expression of the target gene, the transformants were incubated at 37 °C to OD_600_ of 0.6–0.8 and then induced with 1 mM of IPTG at 18 °C for 24 h. Cells were collected and resuspended in lysis buffer (50 mM Tris–HCl; 50 mM NaCl; 1 mM β-mercaptoethanol; 0.1 mM PMSF; 0.5 mM PLP; pH7.0). The samples were ultrasonicated to break the cells. The crude cell lysate was collected after centrifugation at 12,000×g for 30 min. The supernatant was applied to Ni–NTA beads for affinity purification. The column was washed twice with 10 column volume of wash buffer (50 mM Tris–HCl; 50 mM NaCl; 0.5 mM PLP; 20 mM Imidazole, pH7.0). Ten column volume of elution buffer (20 mM Na_3_PO_4_, 0.5 M NaCl; 0.5 mM PLP; 200 mM Imidazole, pH7.0) was used to recover the target protein. The sample was then collected and dialyzed with a Millipore 30-kDa cut-off membrane at 4 °C to remove imidazole and salts, followed by resuspending with storage buffer (50 mM Na_3_PO_4_, 0.15 M NaCl; 0.5 mM PLP; pH7.0). The protein concentration was determined by Bradford Protein Assay Kit (Beyotime).

### The determination of protein concentration by Bradford Assay kit

Dilute BSA gradient with storage buffer of OATA to make the final concentration of BSA 0.125, 0.25, 0.5, 0.75, 1 mg/mL and 1.5 mg/mL, respectively. Take diluted samples (5 μL) to 96 well plates, followed by adding 250 μL G250 staining solution in triplicates for each sample, and then determine the absorbance at OD_595_ with a microplate reader. Take the absorbance value as the vertical axis and the BSA standard protein concentration as the horizontal axis, fit the BSA linear standard curve, calculate the linear regression equation, and then calculate the protein concentration of OATA according to the standard equation.

### Molecular modeling and selecting of mutation sites

The crystal structure of OATA (PDB ID: 5GHF) was used for the molecular simulation. The 1–31 amino acids of chain A in OATA was constructed using the Modeler module (version 9.8) with chain B as the template. Docking simulation was performed with the Discovery Studio (version 20.1.0). Docking simulation with α-ketobutyric acid as the ligand was accomplished using the Flexible Docking (the active site defined by the Binding-Site module, under a default setting of 2000 steps at 700 K for a heating step; 5000 steps at 300 K for a cooling step; 8 Å grid extension). The docking locates most similar to the previous report using pyruvic acid [29] as a ligand was chosen as the docking model.

The active-site models of the L57C/M419I variants were built by amino acid substitution and energy minimization (2000 steps; dielectric constant 4) of the mutation sites.

### Site-directed mutagenesis

To generate each OATA variant, a pair of oligonucleotides including the substitutions (Additional file 1: Table S1) were synthesized and used for reverse PCR with pET28a-OATA as a template (Additional file 2: Figure S1). The PCR product was digested with *Dpn*I, followed by transformation of *E. coli* DH5α. The mutations were identified with DNA sequencing (Sangon, China).

### Analysis of the enzymatic activity of OATA

The enzyme activity was measured with α-ketobutyric acid and isopropylamine as substrates. Briefly, 10 µL of enzyme sample (2.5 mg/mL) was added to 90 µL of reaction mixture containing 300 mM α-ketobutyric acid and 450 mM isopropylamine in 50 mM phosphate buffer (pH7.5), followed by incubating at 37 °C for 30 min. The reaction was terminated with 100 µL of acetonitrile and the sample was centrifuged at 14,000×g for 10 min. The supernatant was diluted 5 times with 400 µL of deionized H_2_O, followed by analysis with HPLC. All experiments were done in triplicate.

### Molecular dynamics simulations of OATAL57C/M419I

The model structure of OATAL57C/M419I was used for molecular dynamics simulations. Molecular dynamics simulations were performed with the YASARA(v18.4.24). Introducing α-ketobutyric acid as a ligand into the receptor was similar to the docking simulation (the active site defined by the Binding-Site module, under a default setting of pH7.5 for the environmental pH.; 8 Å grid extension; energy minimization with the protein).

### Kinetic analysis

To obtain kinetic parameters for α-ketobutyric acid, a pseudo-one-substrate kinetic model was used as described previously [25]. The kinetic parameters were determined from three independent initial rate measurements performed with the same batch of purified enzymes. The concentration of α-ketobutyric acid was 50 to 650 mM while the concentration of isopropylamine was fixed to 1 M. The initial rate was measured by HPLC analysis of produced L-ABA. Initial rate data were fitted to a Michaelis–Menten equation, and the K_m_ and *k*_cat_ values were calculated from the slope and y-intercept of the double-reciprocal plot.

### Screening of OATA mutants with high activity

To prepare the α-ketobutyric acid substrate for the enzyme assay of OATA variants, *E. coli* BL21 (DE3) strain bearing pET28a-TD-His6 was incubated in 200 mL of LB media at 37 °C until OD_600_ reached 0.6–0.8, followed by inducing with 1 mM of IPTG at 18 °C for 12–16 h. The cells were collected and added to 200 mL of reaction mixture containing 300 mM L-threonine and 450 mM isopropylamine in 50 mM of phosphate buffer (pH7.5). The reaction was carried out at 37 °C for 2 h. Thin-layer chromatography (TLC) with Silica TLC gel 60F_10-20 cm_ from Haiyang Chemical Co., Ltd. (Qingdao, China) was performed to monitor the progress of the reaction. After L-threonine was conversed to α-ketobutyric acid, the supernatant was collected by centrifugation at 10,000×g for 10 min. *E. coli* BL21 (DE3) strains bearing various pET28a-OATA-His6 variants were incubated in 8 mL of LB media at 37 °C until OD_600_ reached 0.6–0.8, followed by inducing with 1 mM of IPTG at 18 °C for 14 h. The cells were collected and added to 1 mL of α-ketobutyric acid prepared as above mentioned. The reaction was carried out at 37 °C for 1 h, followed by detection with thin-layer chromatography using Silica TLC gel 60F_10-20 cm_. The solvent system contained A solution and B solution in a ratio of 4:3 (v/v). A solution was 1% (w/v) ninhydrin in N-butanol and B solution was acetic acid in deionized H_2_O (1:2, v/v). After the chromatography, the sildes were dry with a hair drier and the dotes of L-ABA show red color.

### HPLC analysis and chiral analysis of L-ABA

HPLC analysis were performed on a Shimadzu HPLC system (Japan). Analysis of L-ABA and α-ketobutyric acid were performed using YMC-Pack Diol-120-NP column with isocratic elution with 80% Acetonitrile-20% NH_4_H_2_PO_4_ (10 mM, pH 2.0) at 1 mL/min. The column oven temperature was set to 40 °C, UV detection was done at 200 nm (Additional file 1: Figure S3). To analyze the chirality of L-ABA, L-ABA was derivatized using D-glucopyranosyl isothiocyanate (GITC) [30, 31] and detected using a C18 symmetry column (Waters, USA) with 50% methanol and 50% water (containing 0.1%TFA) at a speed of 1 mL/min. The column oven temperature was set to 40 °C. UV detection was done at 254 nm (Additional file 1: Figure S4).

### TD/ω-TA coupled reactions

The coupled reaction for the synthesis of L-ABA was performed at 37 °C with 300 mM L-threonine, 450 mM isopropylamine, 0.5 mM PLP in 50 mM phosphate buffer (pH7.5). The concentrations of TD and OATA were 0.2 mg/mL and 1 mg/mL, respectively. L-ABA was analyzed for measurements of conversion yield and enantiomeric excess.

### Purification of L-2-aminobutyric acid

At the end of the reaction, the reaction solution was heated at 60 °C for 30 min (denature and precipitate the enzymes), followed by centrifuging at 12,000 rpm for 30 min. The supernatant was collected and filtrated by a 0.45-μm filter membrane. The filtrate solution was subjected to cation-exchange by Dowex 50WX8 cation-exchange resin (50 g) in a 50-mL column with rotation for 2 h, followed by washing with 0.1 N HCl (120 mL) and water (120 mL) sequentially. The product was eluted with 145 mL of 10% ammonia solution [12]. The elution was transferred to the rotary evaporator for rotary evaporation at 60–70 °C. After the water was evaporated, the precipitated crystals are washed for three times with ethanol. After each cleaning, L-ABA is obtained by suction filtration.

### Statistical analysis

Experiments were performed in triplicates. The statistical analyses were processed in originlab 2019b, and the structural analysis of OATA was carried out by Discovery Studio 2016 and YASARA (v18.4.24).

## Supplementary Information

**Additional file 1**. Supplementary materials of the full text, including of **Table S1**: The design of primers in six different amino acid sites; **Figure S1:** Thin-layer chromatography (TLC) to evaluate catalytic activities of the mutants for α-ketobutyric acid; **Figure S2**: SDS-PAGE to analyze the purified mutant protein. **Figure S3**: The concentration of L-2-Aminobutyric acid was detected by HPLC; **Figure S4**: Chiral analysis of aminobutyric acid; **Figure S5**: Enzyme kinetic curve of ω-transaminase.

**Additional file 2**. Raw materials of the full text, containing the original, unprocessed and uncropped version of the table 1, Fig 2 and Fig 5 in manuscript. The schematic view of the pET28 plasmid as well as the designed gene structure in assay.

## Data Availability

All data generated or analyzed during this study are included in this published article and its supplementary information/additional files. Sequence data used in this study is deposited in the GenBank under the accession Numbers: WP_011982390.1 (OATA) and WP_000785596.1 (TD).

## Abbreviations

L-ABA: L-2-aminobutyric acid
PLP: Pyridoxal-5′ - phosphate
ω-TA: ω-Transaminase
TD: Threonine deaminase
OATA: ω-Transaminase from *Ochrobactrum anthropi*
TLC: Thin layer chromatography
