# Supplementary material for: Active-site engineering of ω-transaminase from Ochrobactrum anthropi for preparation of L-2-aminobutyric acid
Source: BMC Biotechnol. 2021 Sep 25;21:55. doi: 10.1186/s12896-021-00713-7 (PMC8466713; doi:10.1186/s12896-021-00713-7)
Supplement: Supplementary file 1 — Additional file 1. Supplementary materials of the full text, including of Table S1: The design of primers in six different amino acid sites; Figure S1: Thin-layer chromatography (TLC) to evaluate catalytic activities of the mutants for α-ketobutyric acid; Figure S2: SDS-PAGE to analyze the purified mutant protein. Figure S3: The concentration of L-2-Aminobutyric acid was detected by HPLC; Figure S4: Chiral analysis of aminobutyric acid; Figure S5: Enzyme kinetic curve of ω-transaminase. [file 12896_2021_713_MOESM1_ESM.docx]

**Additional files 1:Table S1.** The design of primers in six different amino acid sites. The wild-type plasmid was used as template for single point saturation mutation. NNN: degenerate primer.

| Primer | Sequence(5**^，^-**3**^，^**) |
| --- | --- |
| Y20-F | taccacttgcactct**NNN**actgacgctgtt |
| Y20-R | gagtgcaagtggtatctgatgtc |
| L57-F | atcgaggctatgtccggt**NNN**tggtccgtt |
| L57-R | accggacatagcctcgatatatc |
| W58-F | gaggctatgtccggtttg**NNN**tccgttggt |
| W58-R | accggacatagcctcgatatatc |
| G229-F | attggtgaaccagttatg**NNN**gctggtggt |
| G229-R | cataactggttcaccaatgaaag |
| A230-F | ggtgaaccagttatgggt**NNN**ggtggtgtt |
| A230-R | cataactggttcaccaatgaaag |
| M419-F | gtgttatctccagagct**NNN**ggtgacactt |
| M419-R | gctctggagataacacctc |


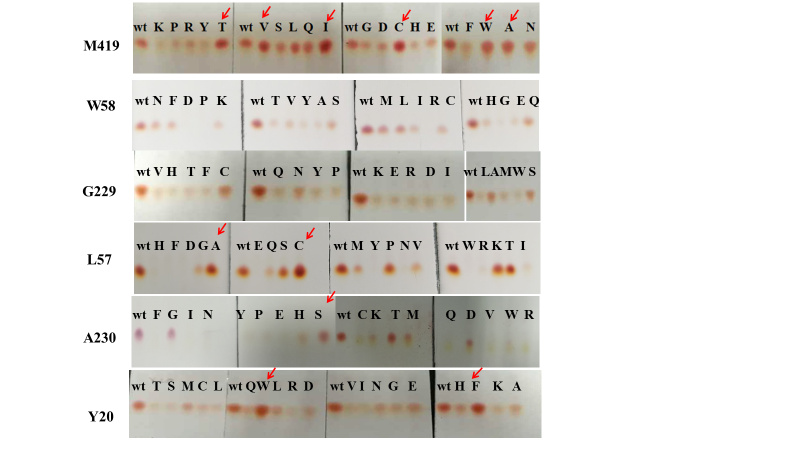


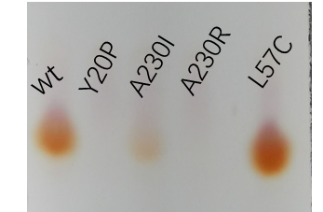


**Additional files 1:Figure S1.** Thin-layer chromatography (TLC) to evaluate catalytic activities of the mutants for α-ketobutyric acid. The mutants with elevated activities were indicated with red arrows. To prepare α-ketobutyric acid substrate for the enzyme assay of OATA variants, E. coli BL21(DE3) strain bearing pET28a-TD-His6 was incubated in 200 mL of LB media at 37°C until OD600 reached 0.6-0.8, followed by inducing with 1 mM of IPTG at 18°C for 12-16 h. The cells were collected and added to 200 mL of reaction mixture containing 300 mM L-threonine and 450 mM isopropylamine in 50 mM of phosphate buffer (pH7.5). The reaction was carried out at 37°C for 2 h. After L-threonine was conversed to α-ketobutyric acid, the supernatant was collected by centrifugation at 10000×g for 10 min. E. coli BL21(DE3) strains bearing various pET28a-OATA-His6 variants were incubated in 8 mL of LB media at 37°C until OD600 reached 0.6-0.8, followed by inducing with 1 mM of IPTG at 18°C for 14 h. The cells were collected and added to 1 mL of α-ketobutyric acid prepared as above mentioned. The reaction was carried out at 37°C for 1 h, followed by detecting with thin-layer chromatography using [Silica TLC](https://www.sigmaaldrich.com/technical-documents/articles/analytical-applications/tlc/unmodified-silica-tlc-plates.html) gel 60F10-20cm.


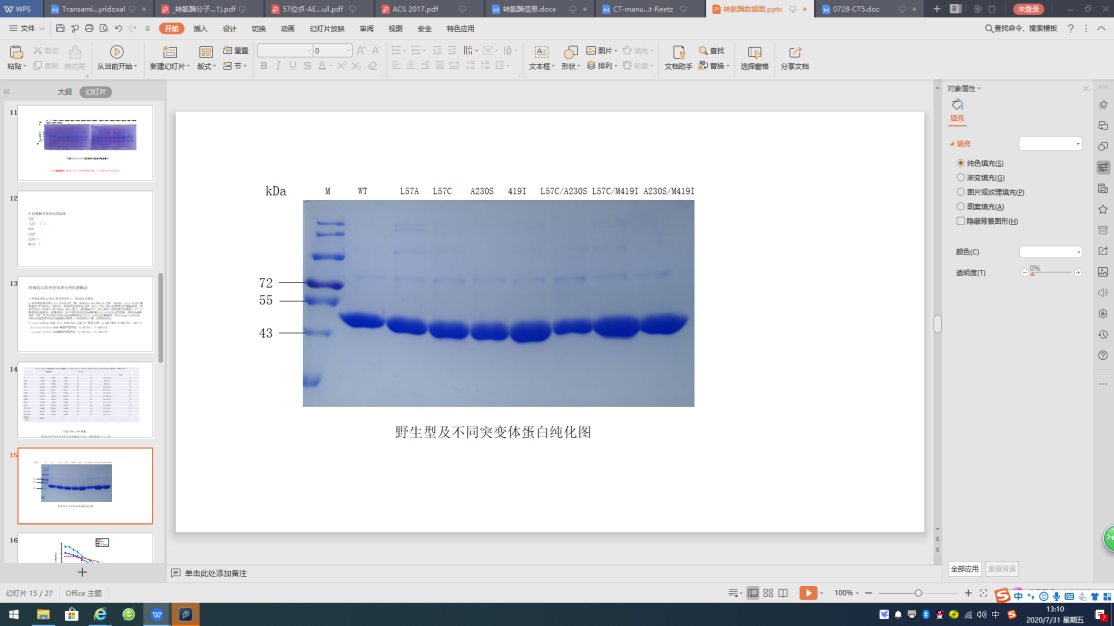


**Additional files 1:Figure S2.** SDS-PAGE to analyze the purified mutant protein. The name of each mutant was indicated on the top of the gel. M: protein molecular weight marker (the size of band was indicated on the left).


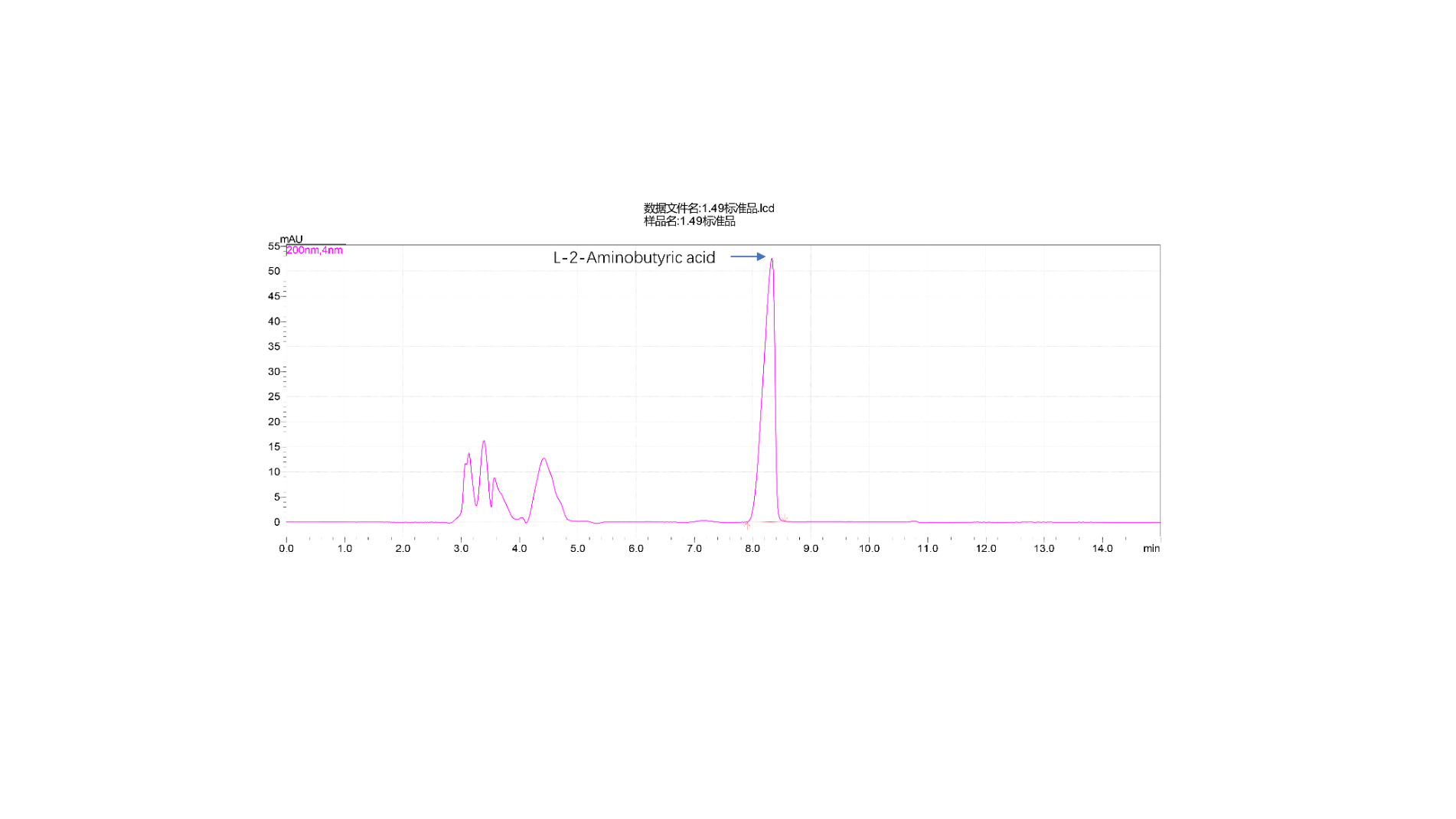


**Additional files 1:Figure S3.** The concentration of L-2-Aminobutyric acid was detected by HPLC. The arrow indicates its peak area.


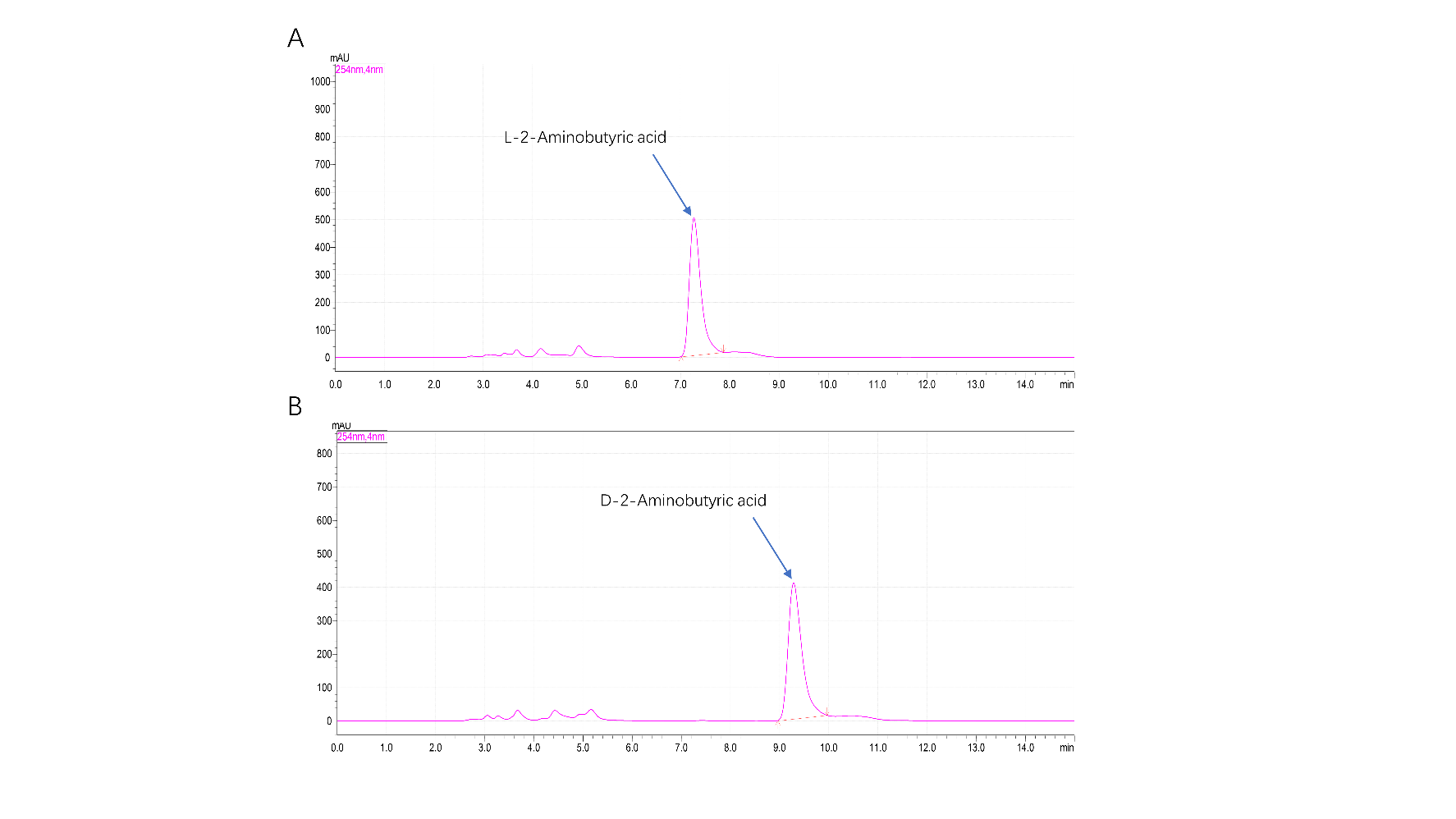


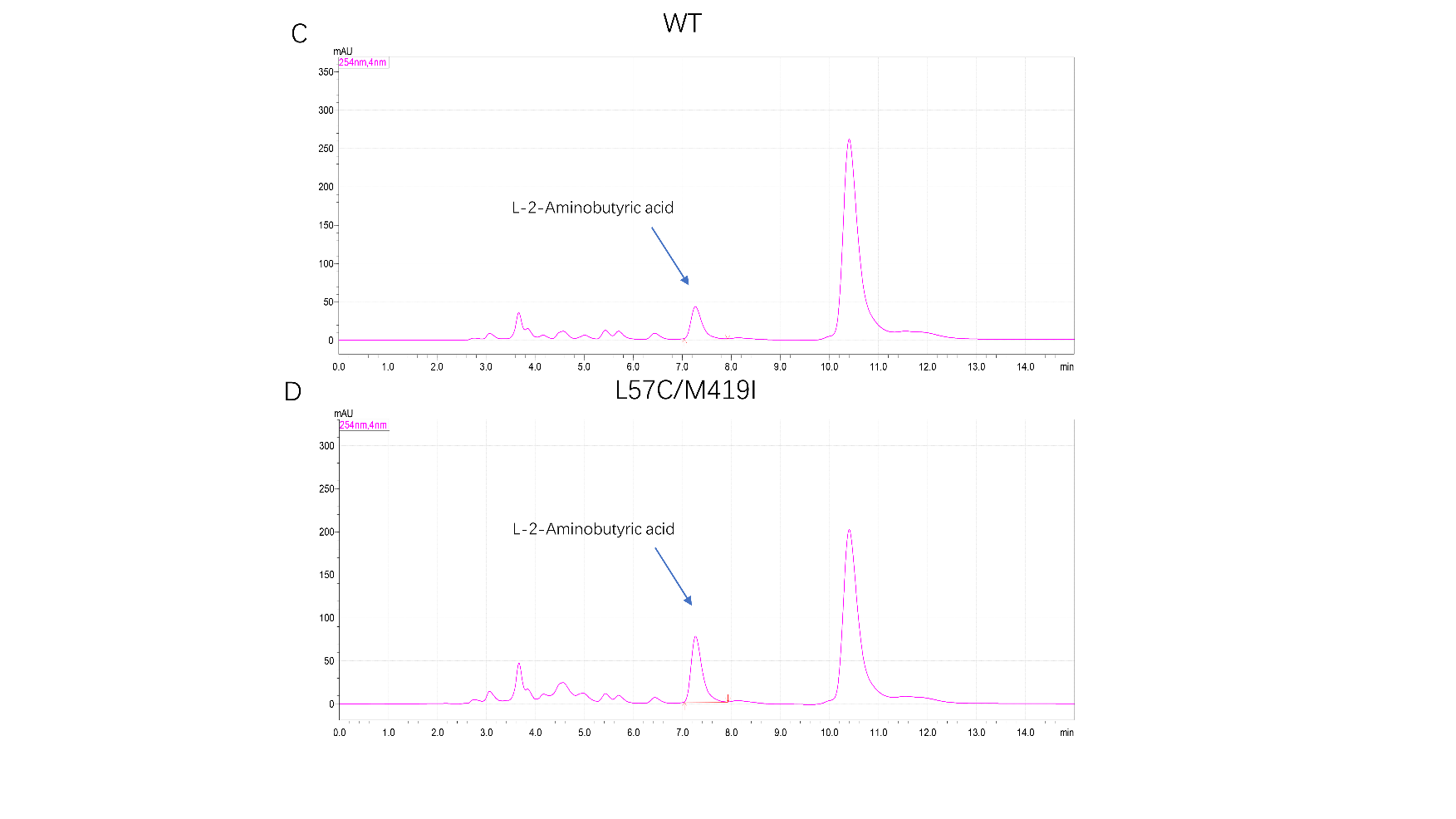


α-ketobutyric acid

**Additional files 1:Figure S4.** Chiral analysis of aminobutyric acid. (A) The standard for L-2-aminobutyric acid. (B) The standard for D-2-aminobutyric acid. (C) and (D) are chiral detection of reaction products of wild-type and L57C/M419I, respectively.


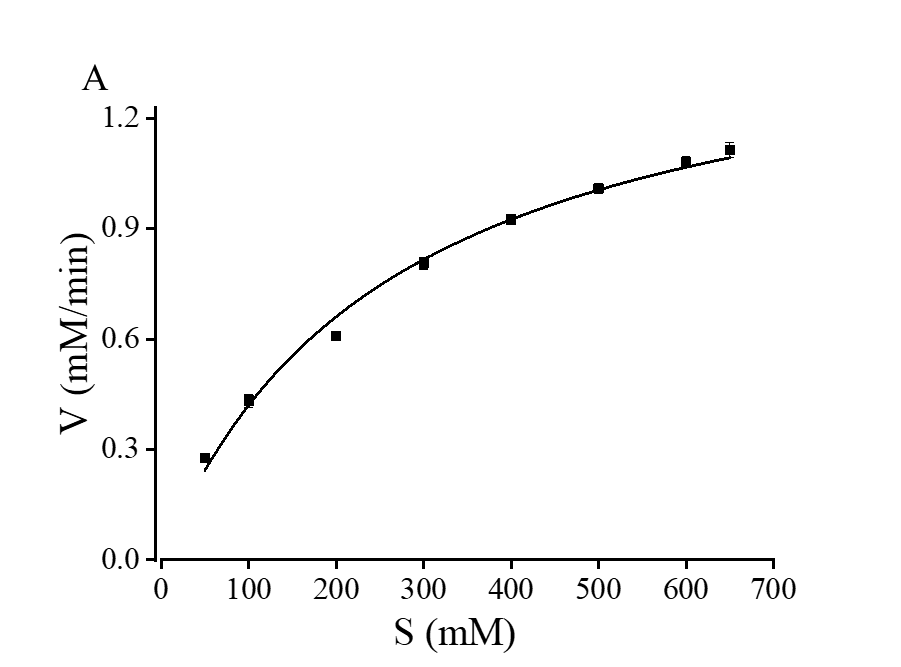
\


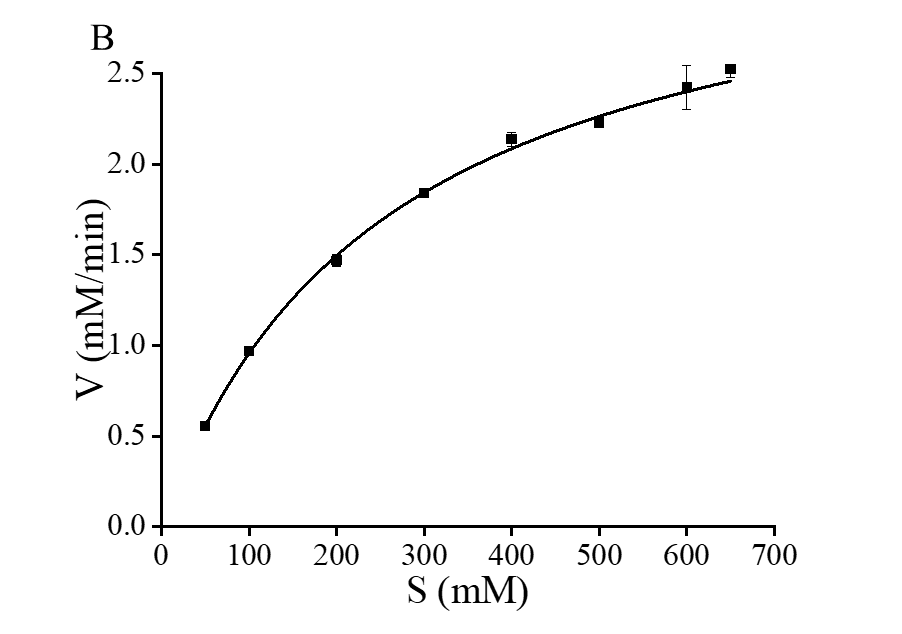


**Additional files 1:Figure S5.** enzyme kinetic curve of ω-transaminase. (A) the wild-type OATA. (B) OATA_L57C/M419I_.The reaction was carried out in 100-μL mixture including 0.5 mM PLP , 0.25 mg/mL OATA, 50-650 mM α-ketobutyric acid at fixed isopropylamine concentration (1 M) with 50 mM phosphate buffer (pH 7.5). The mixture was incubated at 37°C for 30min.
