# Supplementary material for: Active-site engineering of ω-transaminase from Ochrobactrum anthropi for preparation of L-2-aminobutyric acid
Source: BMC Biotechnol. 2021 Sep 25;21:55. doi: 10.1186/s12896-021-00713-7 (PMC8466713; doi:10.1186/s12896-021-00713-7)
Supplement: Supplementary file 2 — Additional file 2. Raw materials of the full text, containing the original, unprocessed and uncropped version of the table 1, Fig 2 and Fig 5 in manuscript. The schematic view of the pET28 plasmid as well as the designed gene structure in assay. [file 12896_2021_713_MOESM2_ESM.docx]

|  | HPLC(Area) |  |  |
| --- | --- | --- | --- |
|  | 1 | 2 | 3 |
| WT | 90257 | 92931 | 93981 |
| L57A | 146002 | 147966 | 145586 |
| L57C | 250519 | 251515 | 251019 |
| A230S | 150874 | 151105 | 154554 |
| M419I | 160677 | 162319 | 165164 |
| L57C/A230S | 108686 | 108152 | 108858 |
| L57C/M419I | 300331 | 290786 | 307475 |
| A230S/M419I | 160402 | 157266 | 158353 |
| Y20W | 88236 | 86583 | 86021 |
| Y20F | 91263 | 90854 | 90721 |
| M419C | 133718 | 125225 | 120171 |
| M419W | 112538 | 126401 | 118362 |
| M419A | 105639 | 93674 | 103826 |
| concentration(1.49g/L) | 880811 |  |  |

**Active-Site Engineering of ω-Transaminase from *Ochrobactrum anthropi* for preparation of L-2-Aminobutyric acid**

Zhiwei Zhang#^[a]^, Yang Liu#^[a]^, Jing Zhao^[a]^, Wenqiang Li^[a]^, Ruiwen Hu^[a]^, Xia Li^[a]^, Aitao Li^[a]^, Yaping Wang*^[a]^, Lixin Ma*^[a]^

[a] Z. Zhang, Y. Liu, Assist. Prof. Dr. J. Zhao, M. Sc. Mol. Biol. & Biochem. W. Li, R. Hu, X. Li, Assist. Prof. Dr. A. Li, Assist. Prof. Dr.YP. Wang, Prof. Dr. L. Ma # These authors contributed equally to this paper *Corresponding author
State Key Laboratory of Biocatalysis and Enzyme Engineering, Hubei Collaborative Innovation Center for Green Transformation of Bio-resources, Hubei Key Laboratory of Industrial Biotechnology, College of Life Sciences, Hubei University 368 Youyi Road, Wuchang Wuhan, 430062, China
E-mail: malixing@hubu.edu.cn, 545080994@qq.com.

**Additional files 2:Table S1.**The sample was diluted 10 times. The area is produced by HPLC.The original, unprocessed and uncropped version of the Fig 2 in manuscript. Comparison of enzyme activities of mutants relative to wild type. The reaction mixture (100 μL) contained 0.5 mM PLP, OATA (0.25 mg/mL), 300 mM α-ketobutyric acid, 450 mM isopropylamine and 50 mM phosphate buffer (pH 7.5). The reaction was carried out at 37°C for 30 min

|  | WT (Area) | | |  | L57C/M419I (Area) | | |
| --- | --- | --- | --- | --- | --- | --- | --- |
| Substrate(mM) | 1 | 2 | 3 | 1 | 2 | 3 |  |
| 50 | 48839 | 48493 | 46748 | 96290 | 95358 | 166689 |  |
| 100 | 78286 | 72340 | 74284 | 170963 | 168543 | 164524 |  |
| 200 | 107154 | 104084 | 105084 | 254541 | 259981 | 248522 |  |
| 300 | 142611 | 137440 | 138356 | 320629 | 318458 | 317229 |  |
| 400 | 159851 | 160199 | 160542 | 373593 | 362480 | 373962 |  |
| 500 | 180625 | 183517 | 185592 | 383407 | 390946 | 382887 |  |
| 600 | 194370 | 199105 | 197958 | 443076 | 403660 | 411695 |  |
| 650 | 197123 | 201393 | 210392 | 429278 | 445295 | 436719 |  |
| Concentration (1.49g/L) | 833745 |  |  |  |  |  |  |

**Additional files 2:Table S2.**The original, unprocessed and uncropped version of the Table 1 in manuscript. Apparent kinetic parameters of the wild-type OATA and OATA_L57C/M419I_ for α-ketobutyric acid. The reaction was carried out in a 100-μL mixture including 0.5 mM PLP , 0.25 mg/mL OATA, 50-650 mM α-ketobutyric acid at a fixed concentration of isopropylamine (1 M) and 50 mM phosphate buffer (pH 7.5). The mixture was incubated at 37°C for 30 min

| WT (Area) | | | L57C/M419I (Area) | | |
| --- | --- | --- | --- | --- | --- |
| 11247 | 14702 | 12373 | 32594 | 29552 | 30418 |
| 25209 | 30274 | 26572 | 46850 | 43987 | 45300 |
| 28030 | 31027 | 29246 | 48556 | 46202 | 47040 |
| 32567 | 36291 | 35113 | 50846 | 48791 | 49760 |
| 37737 | 40189 | 40091 | 52638 | 51262 | 51839 |
| 41846 | 43620 | 42167 | 53513 | 51741 | 51877 |
| Concentration (1.49 g/L) | 833745 |  |  |  |  |

**Additional files 2:Table S3.**The original, unprocessed and uncropped version of the Fig 5 in manuscript. Small-scale preparation of L-ABA with OATA_L57C/M419I_ and TD. The reaction mixture (50 mL) contained 0.5 mM PLP, TD (0.2 mg/mL), OATA (1 mg/mL), 300 mM L-threonine, 450 mM isopropylamine and 50 mM phosphate buffer (pH 7.5). The reaction was carried out at 37°C for 30 min


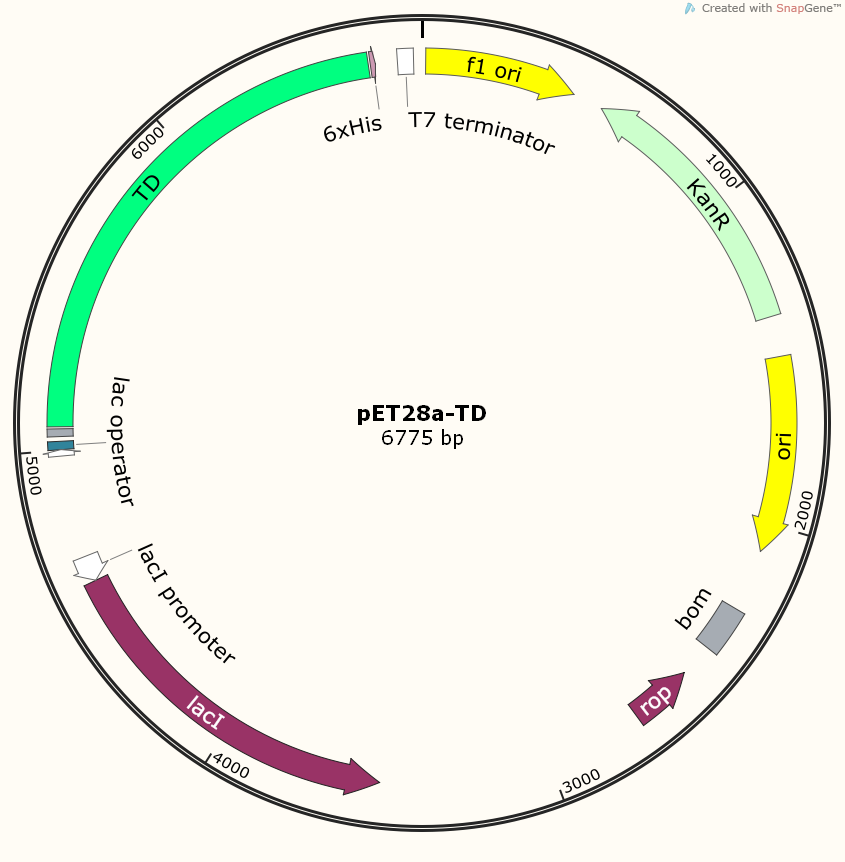

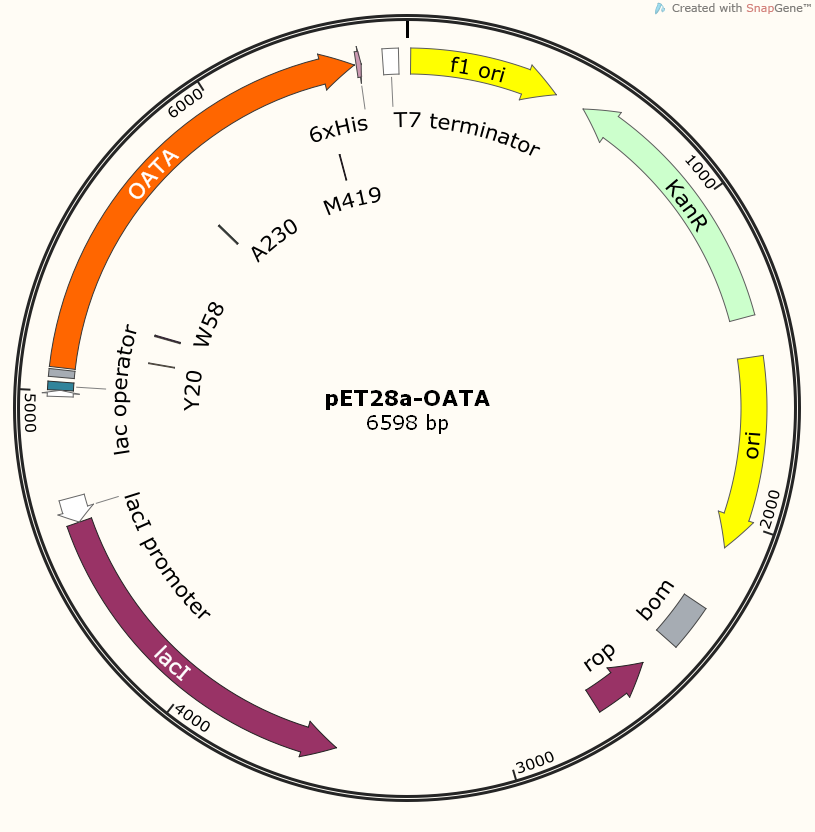


**Additional files 2:Figure S1.**The schematic view of the pET28 plasmid as well as the designed gene structure.
